# Using transfer learning-based plant disease classification and detection for sustainable agriculture

**DOI:** 10.1186/s12870-024-04825-y

**Published:** 2024-02-26

**Authors:** Wasswa Shafik, Ali Tufail, Chandratilak De Silva Liyanage, Rosyzie Anna Awg Haji Mohd Apong

**Affiliations:** School of Digital Science, Universiti Brunei Darussalam, Tungku Link, Gadong, BE1410 Brunei

**Keywords:** Climate action, Convolutional neural networks, Feature extraction, Logistic regression, Plant diseases, Responsible consumption and production, Transfer learning, Zero hunger

## Abstract

Subsistence farmers and global food security depend on sufficient food production, which aligns with the UN's “Zero Hunger,” “Climate Action,” and “Responsible Consumption and Production” sustainable development goals. In addition to already available methods for early disease detection and classification facing overfitting and fine feature extraction complexities during the training process, how early signs of green attacks can be identified or classified remains uncertain. Most pests and disease symptoms are seen in plant leaves and fruits, yet their diagnosis by experts in the laboratory is expensive, tedious, labor-intensive, and time-consuming. Notably, how plant pests and diseases can be appropriately detected and timely prevented is a hotspot paradigm in smart, sustainable agriculture remains unknown. In recent years, deep transfer learning has demonstrated tremendous advances in the recognition accuracy of object detection and image classification systems since these frameworks utilize previously acquired knowledge to solve similar problems more effectively and quickly. Therefore, in this research, we introduce two plant disease detection (PDDNet) models of early fusion (AE) and the lead voting ensemble (LVE) integrated with nine pre-trained convolutional neural networks (CNNs) and fine-tuned by deep feature extraction for efficient plant disease identification and classification. The experiments were carried out on 15 classes of the popular PlantVillage dataset, which has 54,305 image samples of different plant disease species in 38 categories. Hyperparameter fine-tuning was done with popular pre-trained models, including DenseNet201, ResNet101, ResNet50, GoogleNet, AlexNet, ResNet18, EfficientNetB7, NASNetMobile, and ConvNeXtSmall. We test these CNNs on the stated plant disease detection and classification problem, both independently and as part of an ensemble. In the final phase, a logistic regression (LR) classifier is utilized to determine the performance of various CNN model combinations. A comparative analysis was also performed on classifiers, deep learning, the proposed model, and similar state-of-the-art studies. The experiments demonstrated that PDDNet-AE and PDDNet-LVE achieved 96.74% and 97.79%, respectively, compared to current CNNs when tested on several plant diseases, depicting its exceptional robustness and generalization capabilities and mitigating current concerns in plant disease detection and classification.

## Introduction

Agriculture, as a significant driver of the global economy, serves as the primary provider of food, income, revenue, and employment opportunities. Different human societies have been capable of producing food to adequately cater to the current and growing population using advanced technology in the agricultural sector [1]. However, depending on the season or environmental factors, plant pests and diseases are caused by *nematodes*, *fungi*, *viruses*, *protozoa*, and *bacteria* [2, 3]. These severely influence plant health, structure quality, production, quantity, and the economy. One of the highly complex tasks regarding plant protection is the timely identification of plant symptoms, *pests*, and diseases [4]. Traditional approaches used in underdeveloped or developing nations are through human eye inspection, which is inaccurate, tedious, and time-consuming. Furthermore, smart agricultural gadgets are costly, and understanding these obtained classifications and detection on large farms needs agronomists and specialists is expensive [5].

Employing intelligent technologies capable of automatically detecting plant pests and diseases presents a promising approach to reducing total expenses in agriculture [6]. Therefore, academia and industry have used transfer learning (TL) and CNNs, particularly in the agricultural sector, for instance, in plant leaves, fruit, and disease classification, among other applications [7]. However, deep learning (DL) demands an increased number of parameters, thus increasing the training time and resulting in implementing small devices becoming complex and impractical [8]. Furthermore, properly extracting relevant characteristics from any given dataset is vital to the CNN-based model performance; for example, the studies utilized the widely used PlantVillage dataset, with various species of plant diseases across distinct categories [9, 10].

There has been a growing emphasis on rapid plant disease identification and classification using TL architectures. The complexity and required parameters of the TL model are determined by the level of model sophistication and the number of filters utilized [11]. Although TL methods often require advanced image processing techniques, they have simplified the procedure, making it more efficient in terms of time, especially when the model has no starting weights [12]. In addition, TL models require minimal computational resources compared to traditional learning approaches. However, implementing these models on small devices with limited resources can be challenging and limitation of the traditional learning approach [13].

Several studies demonstrate that some current models are developed using the idea of TL to attain better results compared to other well-developed approaches using DL architectures through potent computing equipment, such as graphics processing units (GPUs) and servers [14]. Because of the high cost, it is not practical to use advanced equipment that includes GPUs in the agriculture field that traditional farmers cannot afford. Therefore, there is a need for applications with a reduced number of parameters and reduced levels of computation and power consumption [15].

A survey on adopting computer vision and soft computing methods for disease identification and classification from plant leaves was conducted. It demonstrated that Computer vision techniques enhance plant growth, increasing productivity, quality, and economic value [16]. They are critical in medical, defense, agriculture, remote sensing, and business analysis applications. Digital image processing methods simulate human visual capabilities, providing automatic monitoring, disease management, and water management [17].

Another proposed system used a neural network to segment mango leaves for disease. It involved real-time images, preprocessing, feature extraction, training, and extraction of diseased regions. The system achieved high-level accuracy for anthracnose disease segmentation, with an average Specificity of 0.9115 and Sensitivity of 0.9086. The system demonstrated an intuitive and user-friendly interface and is being developed for precision agriculture [18]. Similarly, a hybrid Fuzzy Competitive Learning-based Counter Propagation Network (FCPN) was proposed for image segmentation of natural scene images. Fuzzy Competitive Learning (FCL) was used to train the instar layer of FCPN, whereas Grossberg learning was used to train the Outstar layer. The region-developing method was utilized for seed point selection, clustering, and estimating the number of crop seeds. The FCPN method produced a lower convergence ratio and greater precision than alternative methods [19].

Pattern recognition and machine vision are indispensable for the resolution of complex problems. Combining conventional and optimization methods, like Nature-Inspired Algorithms (NIA) or Bio-inspired methods, can enhance precision and decrease computational time. One such application is image segmentation, for which the *Bacterial* Foraging Optimization Algorithm (BFOA) is a promising method [20]. The efficiency of the BFO-ANN method was demonstrated through comparison with other approaches. IPM was developed using an automated Radial Basis Function Neural Network (RBFNN) system to detect plant diseases. The system uses leaf images from the IPM agriculture database repository. The RBFNN achieves higher segmentation accuracy than other methods, making it a promising solution for detecting diseases in plants with *biotic* elements [21].

While a considerable number of studies availed some plant disease classification and detection models, there are notable deficiencies in these studies [4, 15, 17, 20], including training on limited dataset size leading to model overfitting and generalization complexity to diverse environments. Training models under controlled backgrounds and environmental conditions, in contrast to the natural setting that makes these models impractical in the natural environment, the accuracy and robustness of models. Computation-related issues, for example, overfitting and difficulties in accurately extracting fine features during training, have impacted the efficiency and usefulness of DL models in the identification and classification of plant diseases. The conventional laboratory diagnosis of plant diseases is expensive, laborious, and time-intensive, which restricts its feasibility for prompt prevention in agriculture. Several current models, like [22, 23], encounter challenges in terms of resilience and ability to apply them to diverse plant diseases since most are only trained on a single crop. Moreover, early classification and detection models proposed were done with restricted image constraints, like images containing colors. However, in the controlled environment, the background and the foreground are put in binary format [24, 25]. However, in this scenario, the approaches employed in many earlier experiments are unsuitable for real-world smart-based agricultural system deployment that employ images that vary with natural-world backdrops.

Such shortcomings leave a gap in the availability of a robust and generalized model trained on the big dataset to detect and classify plant diseases trained on images without restriction on the background to increase the growth truth, thus being practical and implementable on small devices. As illustrated in this study, it employs transfer learning with pre-trained CNNs to improve performance and solve the issue of data scarcity; fine-tuning and deep feature extraction techniques on current cutting-edge CNNs are used to cater to background complexities. Moreover, it tackles computational issues by introducing two models, namely early fusion and lead voting ensemble, that incorporate several pre-trained CNNs; these models assist in overfitting reduction and improving feature extraction.

This study proposes two plant disease detection (PDD) and classification for CNN architecture with considerably reduced parameters. The TL uses nine comparative models: EfficienceNetB7, NASNetMobile, ConvNeXtSmall, DenseNet201, DenseNet101, ResNet50, GoogleNet, ResNet18, and AlexNet. These architectures employ numerous convolutions with changing filter sizes, resulting in superior feature extraction. We have turned to residual connections to address early disease detection problems. We opted for depth-wise separable convolution over conventional convolution because it reduces computational complexity, size, and parameter set without compromising the performance. This study uses a real-time PlantVillage image containing natural image traits. Therefore, this research contributes the following:

- Propose two detection models (PDDNet) CNN architectures integrating the top six common CNNs that extract significant features and perform better. These models are demonstrated concisely. Arithmetic average ensemble (PDDNet-AAE) integrated fine-tuned network outputs. For the use of ensemble feature attraction (PDDNet-AE), the early average fusion method is used. In this instance, we combined deep traits collected from multiple DNNs and then trained with the LR classifier on these combined features. Lead voting ensemble class labels (PDDNet-LVE).
- The study uses a logistic regression classifier to assess the proposed model performance compared to its counterparts (nine pretrained CNNs) that were used to extract deep features. Ultimately, all class labels that were the highest (lead) were voted for, and the system's decision was the most predicted class label.
- The suggested architecture needs minimal parameters and is faster than traditional ML models tested on DenseNet201, DenseNet101, ResNet50, GoogleNet, ResNet18, AlexNet, EfficientNet, NASNet, and ConvNet.
- Since the PlantVillage dataset is the most significant plant disease dataset currently publicly available, it was used to assess the proposed approaches. PDDNet-LVE outperformed the other current network models.
- The proposed models achieved 96.74% and 97.79% accuracy on the early fusion and lead (majority) voting methods for this plant disease detection and classification, respectively. The CNN-LR combination of PDDNet-AE and PDDNet-LVE outperformed the simple averages of CNNs and has demonstrated improved results.

The remainder of this research is arranged into four sections following the introduction. Section "Related Literature" sheds light on the related literature on plant pest and disease classification and detection utilizing TL models, demonstrating the classification techniques used, the type of crop studied, and the reported accuracy. Section "Material and Methods" illustrates the study materials and methodology, including the PlantVillage description and plant diseases within the dataset. Section "Obtained Results and Discussion" demonstrates the results, and related discussions are presented, illustrating performance evaluations on classifiers, sampled deep learning, PDDNet-AE, PDDNet-EA, and PDDNet-LVE, and a comparison based on state-of-art models. Finally, Section "Conclusion" discusses the research conclusion and future research directions.

## Related literature

The section discusses some DL methods for plant pests and disease detection and classification. Traditional ML approaches are based on creating features and segmentation, and DL techniques are based on learning from data in its raw form.

Using pre-trained CNNs like GoogleNet and AlexNet could classify twenty-six pests and diseases within fourteen plant species [7]; 99.34% was obtained through GoogleNet. AlexNet, GoogleNet, VGG, feat, and AlexNetOWTBn could recognize 58 leaf diseases [9]. A nine-layered deep convolutional network was used for plant disease detection, and 96.46% achieved accuracy [26]. Similarly, AlexNet's fully connected layer with GoogLeNet's inception layer to classify four diseases of apple leaves, and the average accuracy score reported 97.62% [27]. For the model optimization, InceptionV3, VGG19, VGG16, and ResNet detected tomato leaf disease and obtained 93.70% field accuracy and 99.60% laboratory accuracy [28]. VGG16 identified eggplant diseases using the super vector machine classifier for red, green, and blue; YCbCr and HSV were tested for robustness with 99.4% RGB [29]. Entirely improved pretrained DL plant disease with 99.75% model classification accuracy.

The authors demonstrated that using the SVM classifier on rice leaf disease classification could categorize eleven deep CNN model features and obtain an average of 98.38% using ResNet50 depth SVM [1]. Authors in [30] identified ten diseases of four *plant species* using six pretrained TL architectures, VGG16 corrected 90% of test datasets, and the authors found three cassava plant diseases and two pest damages using InceptionV3 transfer learning noticed six cassava illnesses using mobile devices [31]. In [32], the study determined that a 50-layer residual neural network can detect three wheat diseases using the ReLU activation and batch normalization following convolution and pooling. Using the German real-time field images, they reported a 96% accuracy. SqueezeNet 227.6MB and SequenzeNet 2.9MB obtained four tea leaf diseases after being tested Cifar ten fast CNN model depthwise separable convolution [33].

A well-trained VGG model identified and classified rice and agricultural diseases [8]. Two inception layers replaced VGGNet's fully connected layers: corn with 80.38% and rice with 92%, respectively. Singh and Misra [34] detailed how the soft computing methods and segmentation of images aid in plant, pest, and disease identification and classification in mostly grown plants like *Malus domestica* (apple), *Zea mays*, and *genus Vitis* diseases using pre-trained CNNs like VGG16 model, some other metaheuristic-inspired algorithms like genetic algorithm.

Gray level co-occurrence matrix (GLCM) with a moveable client-to-server structure for leaf disease detection and their classifications through Gabor wavelet transform (GWT) was used. In the mobile disease diagnosis system, feature vectors represent disease regions that can indicate many resolutions and directions. The mobile client preprocesses leaf photos, *segments*, and the affected leaf sections and sends them to the *Pathology* Server, lowering transmission costs. The Server extracts GWT–GLCM features and classifies K-Nearest Neighbors. Short message service displayed results with 93% accuracy under ideal conditions [35]. Table 1 summarizes the conventional methods, datasets, and the reported performance accuracy corresponding to those methods. In most cases, to summarize this, these studies are presented in three primary stages:

- Plant pests and disease image segmentation is based on applying techniques like mathematical *morphology*, edge detection, color transformation, and pattern classification.
- Detection of plant pests and diseases using traditional ML techniques.
- Representative feature extraction from the segmented images that were obtained utilizing approaches that were based on color, texture, and shape.

**Table 1:** Plant pest and disease literature according to conventional techniques (Note: BPNN, Back Propagation Neural Network; SVM, Support Vector Machine; PNN, Probabilistic Neural Networks)

**Feature extraction approaches**	**Classification techniques**	**Plant pest or disease type**	**Reported accuracy (%)**	**Reference**
Texture features	SVM	Tea	93.33	[36]
Texture and Color features	SVM	Soybean	90.00	[37]
GLCM	SVM	Five leaf disease	95.70	[34]
Gabor wavelets transform and gray-level co-occurrence matrix	KNN		93.0	[35]
Local binary pattern	SVM	Grape	96.60	[38]
color features + GLCM	PNN	Potato	92.00	[39]
Color, shape, and texture feature	Classification tree	Tomatoes	97.30	[40]
Color feature + GLCM	SVM	Grapes	88.89	[41]
Local binary pattern + Zernike moment	SVM	Apples	95.94	[42]
Texture and color and feature	BPNN	Groundnut	97.41	[43]
Color, shape, and texture feature	PNN	Cucumber	91.08	[44]
Scale-invariant feature transforms	SVM	Soybeans	93.79	[45]
Color feature + GLCM method	SVM	Fungal diseases	83.83	[46]
Shape + Color features	–	Paddy	94.70	[47]

The presented models specified in Table 1 are classification algorithms that utilized minimal datasets to differentiate between a limited number of *species*. Some studies used datasets from apple, *Solanum lycopersicum*, *R. groenlandicum*, and maize plants, and most of the reported accuracy ranged from 84% to 97%. Several plant disease detection studies have employed DL as demonstrated. These systems, datasets, and outcomes are demonstrated in Table 2. Most of these experiments included deep network fine-tuning and pretrained CNN feature extraction. To illustrate this, Sabrol and Satish based their study on the tomato disease classification; they used TL to extract features from the images, for example, shape, texture, color, and features with constrained image appearance, and reported a 94% accuracy [40]. The algorithms described in the literature utilize varied datasets and categorize two to four plant species; hence, they cannot be compared directly.

**Table 2:** Plant pest and disease literature according to the conventional techniques

**Classification and Feature extraction techniques**	**Plant pest or disease type**	**Reported accuracy (%)**	**Reference**
SVM classifier +7-layer CNN	Rice	95.48	[48]
Fine tuning GoogLeNet	Plant pest	98.00	[49]
(fine-tuning) DenseNet-121	Apples	92.29	[50]
Improved VGGNet-centred Inception module	Maize	91.83	[51]
Dilated convolution + Inception module	14 different plants	99.37	[52]
Fine-tuning VGG19, ResNet152, DenseNet201, Inceptionv3, and AlexNet	Leaf diseases	93.67	[53]
15-layer CNN architectures	Tomatoes	91.50	[54]
fine-tuning VGG16	Tea	90.00	[55]
(fine-tuning) ResNet50, ResNet152, VGG16, ResNet101, Inceptionv4 and DenseNet121	Plant Leaf diseases	99.75	[56]
9-layer CNNs	Plant Leaf diseases	96.46	[57]
faster R-CNN model	Sugar beets	95.48	[58]
GoogleNet, AlexNet, ResNet, and VGGNet (fine-tuning)	Corns	94.22	[59]
Modified ResNet50	Wheat	98.00	[60]
fine-tuning GoogleNet	Corns	76.00	[61]
13-layer CNN	Soybeans	99.32	[62]
BPNN + GLCM and AlexNet	Leaf diseases	93.85	[63]
7-layer CNN architecture	Rice	95.48	[64]
fine-tuning AlexNet, GoogleNet	Tomatoes	99.18	[65]
ResNet50, VGG19, VGG16, Inceptionv3 (fine-tuning)	Apples	90.40	[66]
SVM Classifier + Inceptionv3	Cassava	93.00	[67]
fine-tuning LeNet	Banana	99.72	[68]
Modified AlexNet	Apples	97.92	[69]
fine-tuning CaffeNet	Leaf diseases	96.30	[70]
Modified VGGNet	Cucumber	82.30	[71]
GoogleNet and AlexNet (fine-tuning)	Leaf diseases	99.35	[72]

## Material and methods

This section entails the background of the deep learning techniques, the PlantVillage dataset, and the proposed methodology.

### Deep learning techniques

Deep learning has been applied extensively in several arenas; its approach to plant disease detection and classification has been extensively used through pretrained deep networks [73–76]. Within this study, we use nine edge-cutting pretrained networks for deep feature extraction for our classification model to have a starting training weight. Table 3 demonstrates the nine pretrained deep CNNs (namely, EfficientNetB7, NASNet, ConvNet, DenseNet201, DenseNet101, ResNet50, GoogleNet, ResNet18, and AlexNet), showing their distinct characteristics on size, accuracy, parameters, depth, and GPU requirements.

**Table 3:** Employed deep network characteristics in this experiment

**Models**	**Size (Megabytes)**	**Parameter (Millions)**	**Depth**	**Input Image Sizes**	**Reference**
DenseNet201	80	20.2	402	224 x 224 x 3	[60]
ResNet101	171	44.7	209	224 x 224 x 3	[67]
ResNet50	98	25.6	107	224 x 224 x 3	[67]
GoogleNet	528	60. 6	22	224 x 224 x 3	[64]
AlexNet	227	61.0	8	227 x 227 x 3	[67]
ResNet18	18	11.7	44	224 x 224 x 3	[60]
EfficientNetB7	256	66.7	438	224 x 224 x 3	[76]
NASNetMobile	23	5.3	389	224 x 224 x 3	[77]
ConvNeXtSmall	192	50.2	28.6	224 x 224 x 3	[78]

### PlantVillage dataset

There is a considerable number of plant pests and disease datasets publicly available, including strawberry [79], rice [80], NLB dataset for maize plant, Turkey-PlantDataset [81], apple, AES-CD9214, PlantVillage, among others. According to the available datasets, we consider the PlantVillage dataset since it has several plant species and over thirty categories with almost all plant characteristics from different datasets. The rest of the datasets checked were found to focus on a single crop that narrows the classification base, and the number of plant leaf images was limitedly low compared to the PlantVillage dataset. Using the pretrained CNNs on a big dataset like PlantVillage assumes proper deep feature extraction. DenseNet models are comparable to ResNet models, except that each layer receives information from all preceding layers. Each Densenet layer feeds forward as early as demonstrated [82]. This study employs DL with six models to extract features to categorize plant diseases. CNNs that have been previously trained and proficient at extracting features and training deep networks. This approach is exceptional since it is more precise in using the LR classifier as a substitute for the output layers of these CNNs.

The PlantVillage dataset was developed to provide effective methods for identifying 38 distinct plant disease classes. It comprises 61,486 plant images in three versions: color, gray-scaled, and segmented. However, we consider 15 categories containing 54,303 PlantVillage images for this experiment. The study considered the PlantVillage dataset with 15 categories since it is more evenly distributed across the different classes than 38 categories. Uneven data distribution can lead to class imbalance issues, where some classes have significantly fewer samples than others. This significantly impacts ML models' performance when accurately predicting the underrepresented classes. Notably, the source of this dataset (https://plantvillage.psu.edu) no longer exists. However, our open-source platforms, including Kaggle and GitHub, have datasets available as linked.

**Fig. 1:**
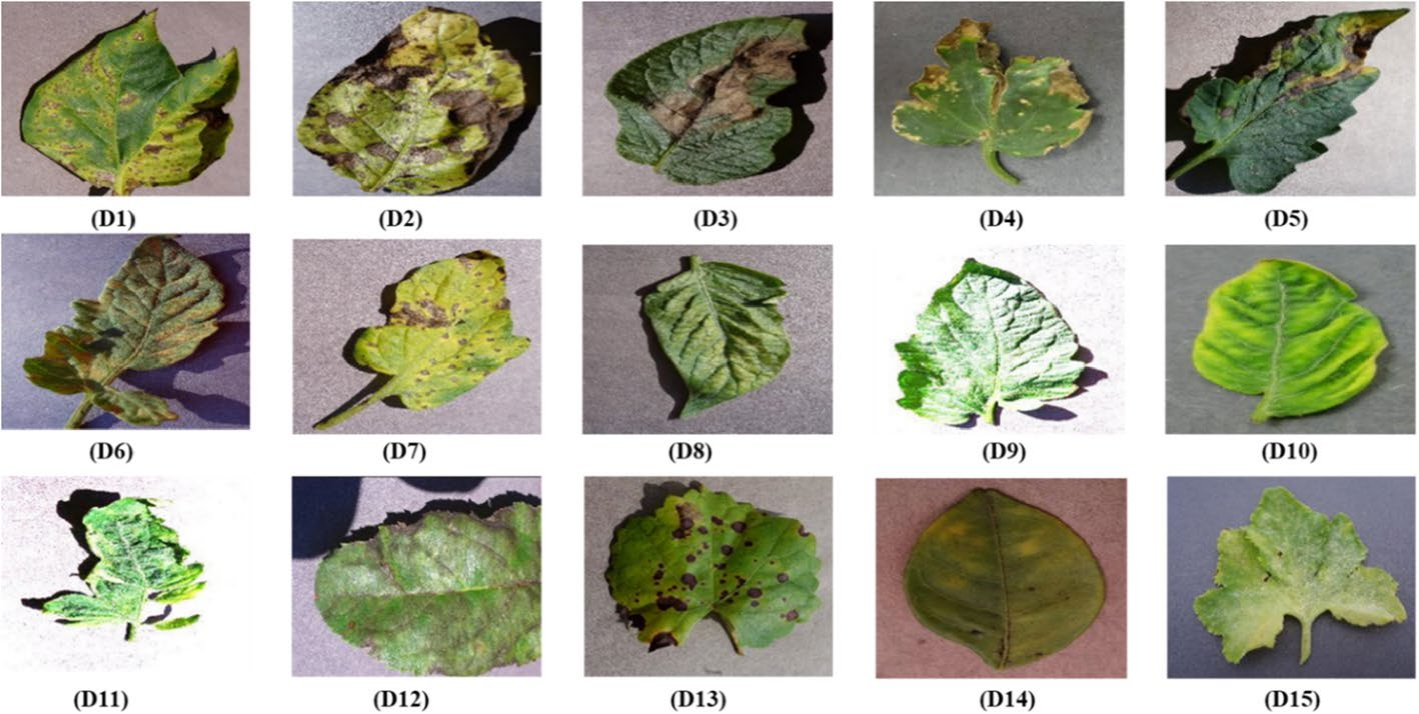
Plantvillage selected leaf image samples from the considered plant dataset in this study. (Legend: D1) Pepper bell *bacterial spot*, D2) potato *early blight*, D3) potato *late blight*, D4) Tomato *bacterial spot*, D5) Tomato *early blight*, D6) Tomato *Lead mold*, D7) Tomato *Septoria leaf spot*, D8) *Spider mites Two-spotted spider mite*, D9) Tomato *target spot*, D10) Tomato *Yellow Leaf Curl Virus*, D11) Tomato *mosaic virus*, D12) Apple *Scrab*, D13) Grape *black rot*, D14) Orange Huanglongbing (Citrus_greening), and D15) Squash *powdery mildew*

Deep features were extracted using nine different pretrained CNNs to make the dataset more diverse and show a wide range of details. During this process, numerous modifications were made employing three channels as well. These enhancements included gamma correction, principal component analysis, noise injection, scaling, image flipping, rotation, and color augmentation. In addition, scaling, rotation, and image flipping (RGB) were used. Figure 1 presents image samples from the PlantVillage plant disease species.

### Methodology

To tackle the challenge of plant disease identification and classification, we consider feature extraction and fine-tuning approaches among the existing TL approaches, including the intermediate layers, fine-tuning, and feature extraction. The selected pre-trained CNNs are used as a feature extractor. The output of the last convolutional layer is used as a feature vector for the new task. Then, the CNNs are fine-tuned on the new dataset. The weights of the lower layers are frozen, and only the weights of the upper layers are updated. TL can save resources, as the model does not need to be trained from scratch.

Therefore, we consider TL for the nine most recent pretrained deep networks: DenseNet201, DenseNet101, ResNet50, ResNet18, GoogleNet, AlexNet, EfficientNetB7, NASNetMobile, and ConvNeXtSmall for feature extraction to aid in the classification problem process. Then, the LR classifier will evaluate the performance at an individual model level, utilizing the weights obtained from these networks. A comparison is then made based on the arithmetic average (AAE), initial (early), amalgamation or fusion (EA), and lead voting ensemble (LVE), commonly referred to as majority voting. Finally, we use the LR classifier to replace a superficial network block for fusion in the PDDNet technique coupled with the final layers of deep neural network in the PDDNet-LVE method.

The image input size is often different depending on the selected pretrained deep network architecture, as the second last column of Table 3 illustrates. For example, AlexNet and DesNet201 require different data inputs of 227 x 227 x 3 and 224 x 224 x 3, among others, at the input layer. Furthermore, due to the diverse CNNs selected for these experiments, the initial convolutional layer and the subsequent convolutional layers use different kernels; for instance, DenseNet201 with all convolutional layers use 3x3 kernels; ResNet101 utilized the initial convolutional layer uses a 7x7 kernel and the all-subsequent convolutional layers use 3x3 kernels.

ResNet50 at the initial convolutional layer uses a 7x7 kernel, and all subsequent convolutional layers use 3x3 kernels. GoogleNet uses 7x7 kernels at the initial convolutional layer uses, and most of the subsequent convolutional layers use 1x1 kernels, while a few layers use 3x3 kernels. AlexNet considers that the initial convolutional layer uses an 11x11 kernel, and the subsequent convolutional layers use 3x3 kernels. Lastly, ResNet18 at the initial convolutional layer uses a 7x7 kernel, and all subsequent convolutional layers use 3x3 kernels, as used during the experiment.

The proposed model approaches were executed using the MATLAB 2022b DL toolbox^1^. The PlantVillage dataset was divided into training, validation, and testing. Adaptive Moment Estimation (Adam) is applied as the optimizer since it employs stochastic optimization, like ML and TL. The recursive nature of the method enables the efficient solving of noisy linear systems and the estimation of extreme values of functions that are only accessible over noisy annotations. Incorporating square propagation in stochastic gradient descent, adaptive gradient, and root mean, Adam combines the benefits of stochastic gradient descent with momentum and root mean square propagation. In addition, the batch sizes varied depending on a step size of 10 within the range of 10 to 100, and it was saturated at 10 epochs. The selected networks were configured with a 1 gradient threshold, and the learning rate ranged between 0.1 to 0.001.

#### PDDNet‑AAE

In this method, we experimented based on an arithmetic ensemble average that included late fusion. Initially, TL was applied to architectures, including DenseNet201, DenseNet101, ResNet50, ResNet18, GoogleNet, AlexNet, EfficientNetB7, NASNetMobile, and ConvNeXtSmall. In this instance, the focal contribution of this study is to substitute the last three layers of these CNNs, that is to say, a fully connected (designed to learn features from the images), a softmax (sometimes called a normalized exponential function that presents covert real numbers to probability function to approximate outcomes), and a classification (follows the softmax layer, it detects, classify mutually exclusive classes (categories) via the cross entropy function) layers with new layer definition. After fine-tuning procedure, the effectiveness of every transfer learning pretrained model was analyzed employing the data prepared for testing. Finally, the results of the PDDNet-AAE ensemble were agreed upon with the rest of the finely adjusted networks.

#### PDDN‑EA

**Fig. 2:**
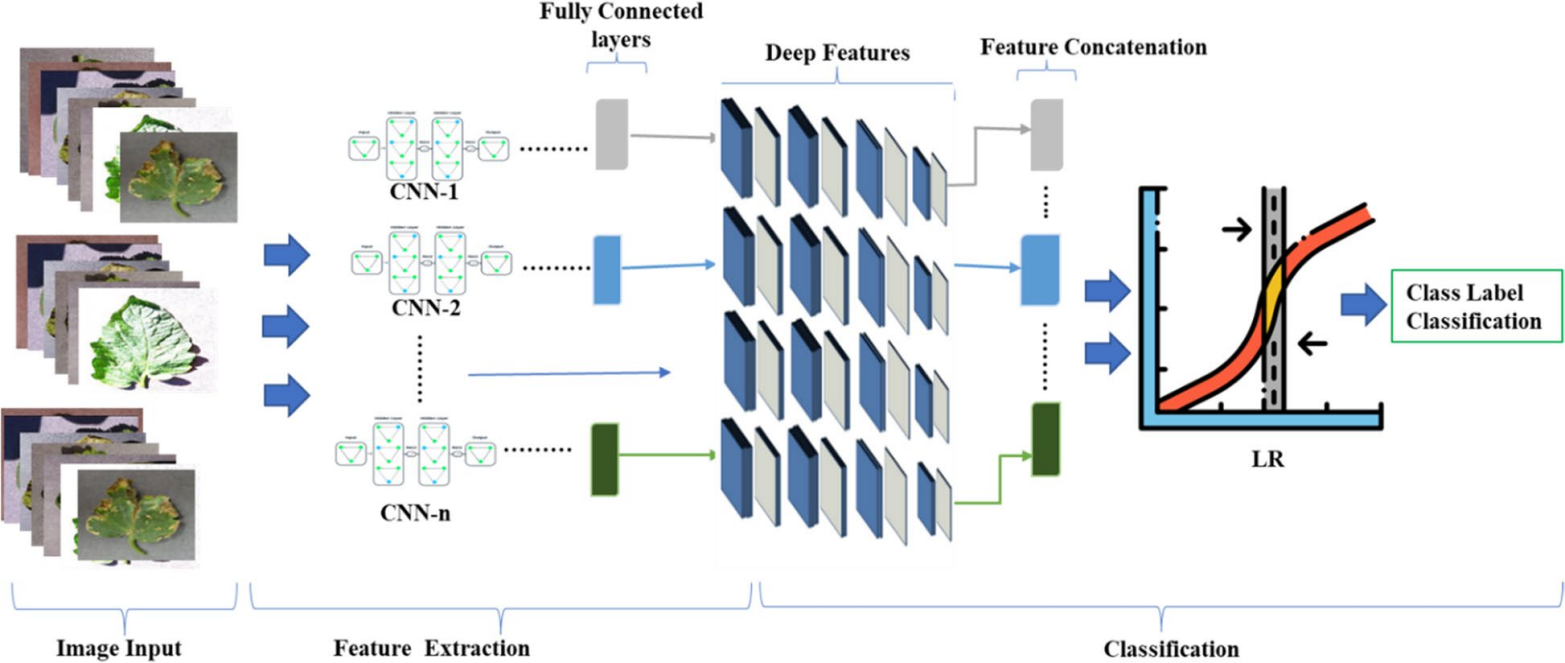
General overview of the PDDNet-EA model

For the early fusion, this model is trained with the LR classifier with features produced from numerous deep networks with fully connected layers and then concatenates these features using the methodology presented (Section "PDDNet‑AAE"). Figure 2 demonstrates an overview of the method's flow diagram.

Considering the demonstrated flow within Fig. 4, the classifier trains the deep features aggregated after being assembled from numerous pretrained networks. Additionally, we employed various combinations of six defined networks to ascertain the class label with the PDDNet model that we suggested. It is significant to mention that these pretrained networks were utilized in this ensemble.

#### PDDNet‑LVE

**Fig. 3:**
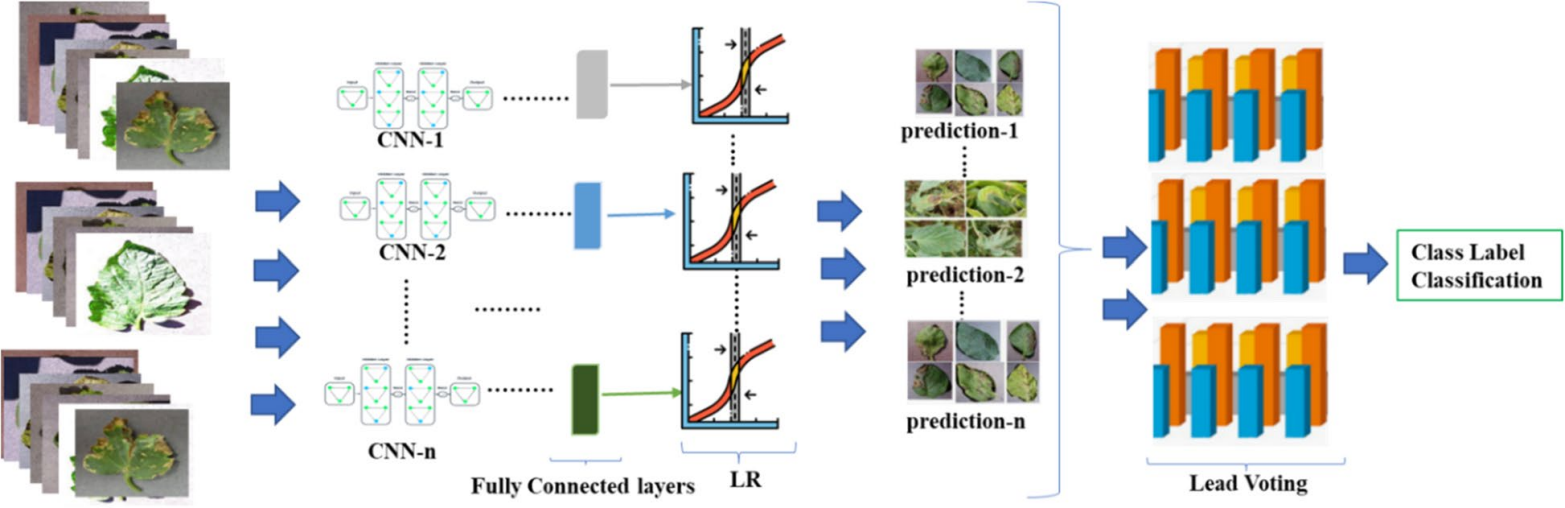
General overview of the PDDNet-LVE model

We started by extracting deep features from the layers of these fully connected architectures. Then, the final three layers were changed to the LR classifier of previously trained deep network architectures. The deep features accumulated from every architecture were utilized during classifier training. Finally, the approach of lead voting by a majority (LVE) was employed for all existing labels within the PlantVillage dataset. Only the class label considered to have the highest level of accuracy served as the final selection for the method (LVE), as depicted in Fig. 3.

## Obtained results and discussion

This section mainly demonstrates the obtained results and the corresponding discussions of proposed models of an integrated ensemble LR model classifier that uses deep features and averages of the CNN models. The proposed models are based on deep feature extraction, and then we tested three model approaches, namely AAE, EA, and LVE, employing pretrained networks.

We used the PlantVillage dataset to test the suggested approach described in subsection "Methodology". This dataset includes color, gray-scaled, and segmented image categories, encompassing healthy and unhealthy plant left species collected and utilized in their natural ecological setting. Table 4 provides the dataset class literature according to disease names. Table 5 depicts the plant type and image sampling quantities used in this research's training and testing phases; the computer and simulation parameters are presented in Table 6.

**Table 4:** PlantVillage dataset based on the disease names

**Plant Class**	**Affected Plant**	**Disease names**	**Reported samples**		**Total Images**
			**Training**	**Testing**	
CD1	Tomatoes	Tomato Yellow Leaf Curl Virus	4,286	1071	5,357
CD2	Tomatoes	Tomato mosaic virus	299	74	373
CD3	Tomatoes	Target spot	1,123	281	1,404
CD4	Tomatoes	Spider mites Two-spotted spider mites	1,341	335	1,676
CD5	Tomatoes	Septoria leaf spot	1,417	354	1,771
CD6	Squash, Cherry	Powdery mildew	2,310	577	2,887
CD7	Corn	Northern Leaf blight	817	197	1,014
CD8	Strawberry	Leaf scorch	887	222	1,109
CD9	Tomato	Leaf mold	761	191	952
CD10	Grapes	Leaf blight Isariopsis Leaf Spots	861	215	1,076
CD11	Tomato, Potato	Late blight	2327	582	2,909
CD12	Oranges	Haunglongbing Citrus greening	4,405	1,102	5,507
CD13	Grapes	Esca black measles	1,107	276	1,383
CD14	Tomatoes, Potatoes	Early blight	1600	400	2,000
CD15	Corn	Ordinary rust	953	239	1,192
CD16	Corn	Cercospora leaf spot gray leaf spot	440	103	543
CD17	Apples	Cedar apple rust	220	55	275
CD18	Apples, Grapes	Black rots	1,140	361	1501
CD19	Tomatoes, bell, Pepper, and Peach	Bacterial spot	4,337	1,084	5,421
CD20	Apple	Apple *scab*	504	126	630
CD21	Tomatoes	Healthy	4,909	1,200	6,109

**Table 5:** PlantVillage dataset based on plant type

**Plant Class**	**Affected Plant**	**Disease name (as per dataset)**	**Reported samples**		**Total images**
			**Training**	**Testing**	
Tomato Class	Tomatoes	Tomato Yellow Leaf Curl Virus	4,286	1,071	5357
	Tomatoes	Tomato mosaic virus	299	74	373
	Tomatoes	Target spot	1,123	281	1,407
	Tomatoes	Spider mites Two-spotted spider mites	1,341	335	1,676
	Tomatoes	Septoria leaf spot	1,417	354	1,771
	Tomatoes	Leaf mold	761	191	952
	Tomatoes	Late blight	1,527	382	1,909
	Tomatoes	Early blight	800	200	1,000
	Tomatoes	Bacterial spots	1,702	425	2,127
	Tomatoes	Healthy	1,273	318	1,591
Maize Class	Maize Corn	Northern Leaf Blight	788	197	985
	Maize Corn	Ordinary rust	953	239	1,192
	Maize	Cercospora leaf spot gray leaf spot	410	103	513
	Maize Corn	Healthy	929	233	1,162
Apple Class	Apples	Cedar apple rust	220	55	275
	Apples	Black rots	496	125	621
	Apple	Apple cab	504	126	630
	Apple	Healthy	1,316	329	1,645
Potato Class	Potatoes	Late blight	800	200	1,000
	Potatoes	Healthy	121	31	152
	Potatoes	Early blight	800	200	1,000
Strawberry Class	Strawberries	Healthy	364	92	456
	Strawberries	Leaf scorch	887	222	1,109
Squash Class	Squashes	Powdery mildew	1,468	367	1,835
Soybean Class	Soybeans	Healthy	4,072	1,018	5,090
Raspberry Class	Raspberry	Healthy	297	74	371
Pepper Class	Pepper bells	Healthy	1,183	295	1,478
	Pepper bells	Bacterial spots	797	200	997
Peach Class	Peaches	Healthy	288	72	360
	Peach	Bacterial spot	1,838	459	2,297
Orange Class	Orange	Huanglongbing	4,405	1,102	5,507
Grape Class	Grape	Leaf blight	861	215	1,076
	Grape	Healthy	339	84	423
	Grape	Esca	1,107	276	1,383
	Grape	Black rot	944	236	1,180
Blueberry Class	Blueberry	Healthy	1,202	300	1,502
Cherry Class	Cherry	Powdery mildew	842	210	1,052
	Cherry	Healthy	684	170	854

**Table 6:** Accuracy performance for every class using different classifiers (for plant class identifier, consider Fig. 1 for details) with the proposed model

Plant Class	PDDNet‑EA model (%)					PDDNet‑LVE Model (%)				
identifier	NB	LR	RF	KNN	SVM	NB	LR	RF	KNN	SVM
D1	0.52	0.76	0.49	0.7	0.75	0.44	1	0.78	0.96	1
D2	0.57	0.9	0.67	0.79	0.87	0.57	0.91	0.8	0.83	0.9
D3	0.7	0.95	0.56	0.81	0.93	0.42	0.98	0.87	0.95	0.95
D4	0.79	**1**	0.57	0.91	0.94	0.49	0.9	0.89	0.84	0.89
D5	0.82	**1**	0.61	0.93	0.95	0.53	0.97	0.72	0.94	0.94
D6	0.97	0.99	0.69	0.98	0.99	0.64	0.79	0.63	0.62	0.75
D7	0.96	**1**	0.66	0.99	**1**	0.56	0.95	0.86	0.87	0.91
D8	0.67	0.81	0.73	0.79	0.83	0.71	0.83	0.82	0.65	0.78
D9	0.72	0.89	0.67	0.83	0.89	0.57	0.99	0.63	0.98	0.96
D10	0.62	0.79	0.61	0.7	0.81	0.43	**1**	0.81	0.96	0.99
D11	0.76	0.91	0.68	0.86	0.9	0.61	0.97	0.62	0.76	0.94
D12	0.7	0.94	0.89	0.91	0.93	0.87	**1**	0.78	0.95	0.99
D13	0.69	0.96	0.72	0.92	0.96	0.62	0.95	0.53	0.73	0.92
D14	0.61	0.93	0.87	0.88	0.94	0.81	0.82	0.71	0.66	0.82
D15	0.71	0.99	0.75	0.94	0.99	0.69	0.88	0.57	0.69	0.83

We discuss the results and performance assessments in detail in the following subsections. The experiments were used using Matlab2022b simulator^2^ and NVIDIA^3^ with GeForce RTX 2070 and DirectX runtime version 12.0.

### Performance evaluation of classifiers

There are five standard classifiers, for instance, K-Nearest Neighbor (KNN), Support Vector Machine (SVM), Random Forest (RF), LR, and Naive Bayes (NB), that are often used in deep learning methodologies to evaluate these pretrained CNNs networks. Table 7 illustrates the testing accuracy for every class using different classifiers. Moreover, the model performances are further assessed in terms of F1 scores, accuracy, recall, and precision using False Positives (FP), False Negatives (FN), True Negatives (TN), and True Positives (TP).

**Table 7:** Performance assessment of PDDNet‑EA and PDDNet‑LVE models

**Performance Criteria**	**PDDNet‑EA model (%)**					**PDDNet‑LVE Model (%)**				
identifier	NB	LR	RF	KNN	SVM	NB	LR	RF	KNN	SVM
F1- scores	0.685	0.942	0.771	0.853	0.913	0.684	0.944	0.774	0.856	0.916
Recall	0.670	0.967	0.793	0.876	0.928	0.672	0.968	0.795	0.877	0.926
Precision	0.932	0.975	0.932	0.839	0.908	0.934	0.977	0.930	0.840	0.909

TP represents the number of instances correctly predicted as positive by the model. In other words, it corresponds to the case where the model predicts the positive class correctly. TN represents the number of instances correctly predicted as negative by the proposed model. It corresponds to the case where the model predicted the negative class correctly. FP epitomizes the number of instances incorrectly predicted as positive by the model. It corresponds to the case where the model predicted the positive class when the actual class was negative. Finally, FN denotes the number of instances incorrectly predicted as negative by the model. It corresponds to the case where the model predicted the negative class when the actual class was positive.

#### Accuracy

The term "accuracy" is the proportion of correct predictions completed compared to the total number of data points collected (T). In scientific literature, it is referred to as recognition, correctness, or success rate and expressed as Eq. 1.

1 $$\mathrm{Accuracy }=({\text{TN}}+{\text{TP}})/{\text{T}}$$

#### Precision

The proportion of actual positive samples found to the total samples anticipated to be positive calculated as presented in Eq. 2.

2 $$\mathrm{Precision }={\text{TP}}/({\text{TP}}+{\text{FP}})$$

#### Sensitivity and recall

The term "sensitivity" or "recall" refers to the proportion of correctly anticipated positives to the total number of actual positive results (Eq. 3).

3 $$\mathrm{Recall }={\text{TP}}/({\text{FN}}+{\text{TP}})$$

#### F1- scores

The F1-score refers to the harmonic mean of precision and sensitivity (recall), expressed in Eq. 4.

4 $${\text{F}}1 =2*\left({\text{Recall}}*{\text{Precision}}\right)/\left({\text{Recall}}+{\text{Precision}}\right)$$

Based on the testing accuracies presented in Table 6, on average, LR obtained 93.88 %, NB with 70.98%, KNN with 84.93 %, SVM with 91.55%, and RF with an accuracy performance of 78.43%, thus making us select LR to be used during the experiments leading to the conclusion that increasing the data size improves exceptionally the performance accuracies. Table 7 presents the precision and recall values and F1 scores. Finally, Table 8 illustrates the accuracy scores obtained with different batch sizes in the LR classifier.

**Table 8:** Accuracy scores obtained with different batch sizes in the LR classifier

**Identifiers**	**PDDNet‑EA model (%)**					**PDDNet‑LVE Model (%)**				
Batch sizes	16	32	48	64	80	16	32	48	64	80
Accuracies	93.83	90.53	88.3	86.44	85.8	93.83	90.73	88.1	86.48	85.9

### Performance evaluation on deep learning

We performed fine-tuning for previously trained CNN models using the DL methodology to evaluate these DL networks. The process of fine-tuning was accomplished by transferring new layers to our plant disease detection and classification problem to replace the deep CNN's last three layers, as described earlier. We examined the accuracy of fine-tuning to observe the effect of TL on the overall performance of the counterparts. After using the hyperparameter fine turning, we considered the minimum batch capacity to be sixteen, the max epochs were put to 10, 0.0001 on the weight decay adjustments, and the learning rate primarily ranged from 0.001 to 0.01. Similarly, for the learning optimization approach, a Mini Batch Stochastic Gradient Descent (MB-SGD) was applied for the deep neural networks to optimize their performance. As a result, 5000 iterations were fully completed for the training procedure, and the obtained accuracies are presented in Table 9. The bold figures within all tables denote the best-performing model.

**Table 9:** Fine-tuned accuracy scores of pretrained networks in percentages

**Models**	**Accuracy scores (%)**
DenseNet201	**93.48**
ResNet101	93.25
ResNet50	93.03
ResNet18	91.45
GoogleNet	87.62
AlexNet	86.93
EfficientNet	93.16
NASNet	92.6
ConvNet	92.90

According to Table 9, the DenseNet201 achieved the highest accuracy among pretrained models based on transfer learning, achieving 93.48%, while the AlexNet achieved the lowest performance with 86.93%. Both results can be compared to those attained using transfer learning on the DenseNet201 architecture. It is further observed that an increment in the complexity improves the accuracies. According to these reported results, the last layer of these models is replaced with the LR classifier. Consequently, the LR was fed with deep features extracted from pretrained CNN networks, presented in Table 10.

**Table 10:** Obtained accuracy after replacing the last layer with a linear regression (LR) classifier

**Models**	**Accuracy scores (%)**
DenseNet201	**94.86**
ResNet101	94.64
ResNet50	94.21
ResNet18	91.84
GoogleNet	90.89
AlexNet	90.40
EfficientNet	94.57
NASNet	92.9
ConvNet	93.10

The LR classifier parameters used were quadratic kernel functions, cubic and tenfold cross-validation approach, and the "one versus all" strategy, which was proven to be the most effective evaluator. According to Table 11, the DenseNet201 model demonstrated an accuracy of 94.86% when detecting plant diseases. Depending on the results, this was the maximum level of accuracy that could be attained after several fine turns. More interestingly, the presented findings in Table 9 are improved to those in Table 10, demonstrating that utilizing the LR as the last layer is advantageous. As a result, we use LR with the other pretrained models with deep features for the remaining part of the experiments.

**Table 11:** Accuracy of the final fusion centered on fine-tuned DNs (refer to Fig. 3 for disease numbering)

**Disease number**	**Plant Disease Specification**	**Accuracy scores (%)**
D1	Pepper bell bacterial spot	92.85
D2	Potato early blight	83.3
D3	Potato late blight	90.1
D4	Tomato bacterial spot	89
D5	Tomato early blight	99.32
D6	Tomato Lead mold	85.95
D7	Tomato Septoria leaf spot	86.15
D8	Spider mites Two-spotted spider mites	**100**
D9	Tomato target spot	85.7
D10	Tomato Yellow Leaf Curl Virus	96.88
D11	Tomato mosaic virus	98.6
D12	Apple Scrab	**100**
D13	Grape black rot	99.1
D14	Orange Haunglongbing	**100**
D15	Squash powdery mildew	80.65

### Performance evaluation on PDDNet‑ AAE model

To evaluate this proposed model, a combination of the above-mentioned pretrained CNNs is used by calculating the average scores from these networks for each class as early as demonstrated in [64]. The accuracy score was calculated using the score-based fusion technique of the deep CNNs with the finest performance, as Table 11 demonstrates. Based on the class distribution, the weighted average accuracy was 93.7%.

### Performance evaluation on the PDDNet‑EA model

The early fusion that was hypothesized, the CNN-LR model, was initially developed based on an early fusion combining the information gathered from the deep CNNs (as Fig. 2 demonstrated). Through several combinations of the six selected CNNs, we achieved the outcomes provided in Table 12 in the subsequent columns, determined by the average accuracy and the standard deviation of those scores. For example, based on Table 13, the PDD-AAE model's maximum accuracy score was 96.79% using DenseNet201, ResNet101, AlexNet, ResNet50, and GoogleNet networks. Because of this, utilizing a pretrained version of ResNet18 in the presence of ResNet50 and ResNet101 is not productive, as most networks provide the most significant results without being used.

**Table 12:** The PDDNet-EA and PDDNet-LVE model results (σ = standard deviation)

**Combination**	**PDDNet-EA model**			**PDDNet-LVE model**		
	**F1-Score (%)**	**Accuracy (%)**	**σ**	**F1-Score (%)**	**Accuracy (%)**	**σ**
ResNet101 + ResNet50 + DenseNet201 + GoogleNet + AlexNet	95.02	**96.94**	0.1569	97.07	**97.79**	0.2431
ResNet50 +ResNet101+ AlexNet + GoogleNet + ResNet18 DenseNet201	95.75	96.83	0.1175	96.81	97.21	0.1203
ResNet101+ AlexNet + ResNet50+ ResNet18+ DenseNet201	95.52	96.78	0.1537	96.61	96.99	0.0828
ResNet101+ResNet50 + ResNet18+ DenseNet201+ GoogleNet	95.67	96.67	0.1614	96.29	96.90	0.1289
EfficientNetB7+ NASNetMobile+ ConvNeXtSmall+ AlexNet	95.78	96.92	0.1614	96.89	97.80	0.1299
DenseNet201+ResNet101+ GoogleNet+ AlexNet	95.96	96.58	0.1305	96.02	96.65	0.1293
ResNet50+ DenseNet201+ GoogleNet + AlexNet	95.77	96.56	0.1013	95.95	96.58	0.1071
ResNet101+ ResNet50+ GoogleNet + AlexNet	95.81	96.45	0.2089	95.72	96.42	0.1654
DenseNet201+ ResNet101+ ResNet50	96.15	96.42	0.1062	96.45	95.75	0.1383

**Table 13:** Comparison of proposed network models on the accuracy scores with pretrained networks

**Models**	**Accuracy scores (%)**
DenseNet201	93.48
ResNet101	93.25
ResNet50	93.03
ResNet18	91.45
GoogleNet	87.62
AlexNet	86.93
EfficientNet	93.16
NASNet	92.6
ConvNet	92.90
PDDNet-EA	**96.94**
PDDNet-LAE	**97.79**

### Performance evaluation on the PDDNet‑LVE model

The results were produced with the PDDNet-LVE model, based on the lead (majority) votes obtained from detecting the class labels acquired from the LR classifier with deep features presented in Fig. 3 and the last column of Table 12. Moreover, the maximum accuracy score possible with the PDD-LVE model was attained when a mixture of AlexNet, DenseNet201, ResNet50, ResNet101, and GoogleNet was used. This resulted in accuracy scores of 96.94% and 97.79% for the EA and LVE models, respectively. These findings are consistent with those seen in Table 13, which shows that the best outcomes were achieved with all CNNs.

### Comparison with edge-cutting models

As demonstrated earlier, CNNs have widely been used in object class label classification, object recognition patterns, and objection detection most recently. Since the most pretrained deep networks were considered, DenseNet201, DenseNet101, ResNet50, GoogleNet, ResNet18, AlexNet, EfficientNetB7, NASNetMobile, and ConvNet have been compared based on the documented accuracy with the most recent published results about plant disease classification [83]. Table 13 demonstrates the accuracy of the models used during the experiment, and Table 14 shows the recently proposed model using some or all used pretrained models during the study.

**Table 14:** Comparison of proposed network models on the accuracy scores with pretrained networks

**Reference**	**Year of Publication**	**Classification and Feature extraction techniques**	**Plant pest or disease type**	**Reported accuracy (%)**
[70]	2016	fine-tuned DenseNet201+Inceptionv3+ResNet152+ VGG19 and AlexNet	Leaf diseases	93.67
[54]	2019	15-layer CNN	Tomato	91.50
[48]	2019	SVM classifier + 7-layer	Rice	95.48
[50]	2020	fine-tuned DenseNet-121	Apples	92.29
[51]	2023	Enhanced VGGNet-based Inception module	Potatoes	91.83
[84]	2023	SVM classifier + CNN	Paddy	91.45
[85]	2023	SMoGW-DCNN	Leaf diseases	94.5
[86]	2023	GP2D2	Paddy	89.4
[87]	2023	Inception V3 model + Adam Optimizer	Basil and Mint Leaves	70.89
Proposed model		PDDNet-EA	PlantVillage	**96.94**
		PDDNet-LAE	PlantVillage	**97.79**

**Fig. 4:**
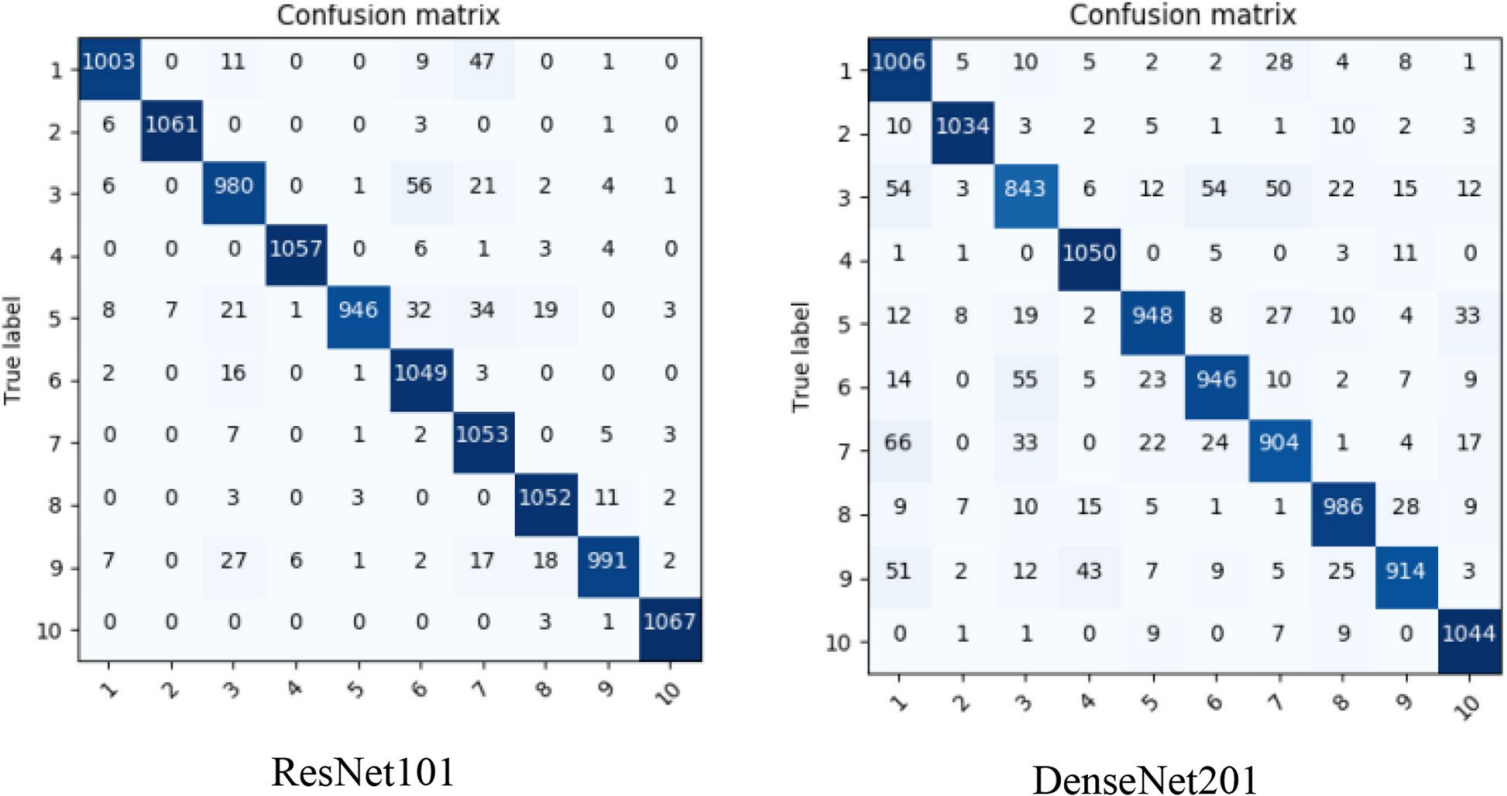
Tomato leaves (PlantVillage) classification results of the best performed amongst the selected CNNs

**Fig. 5:**
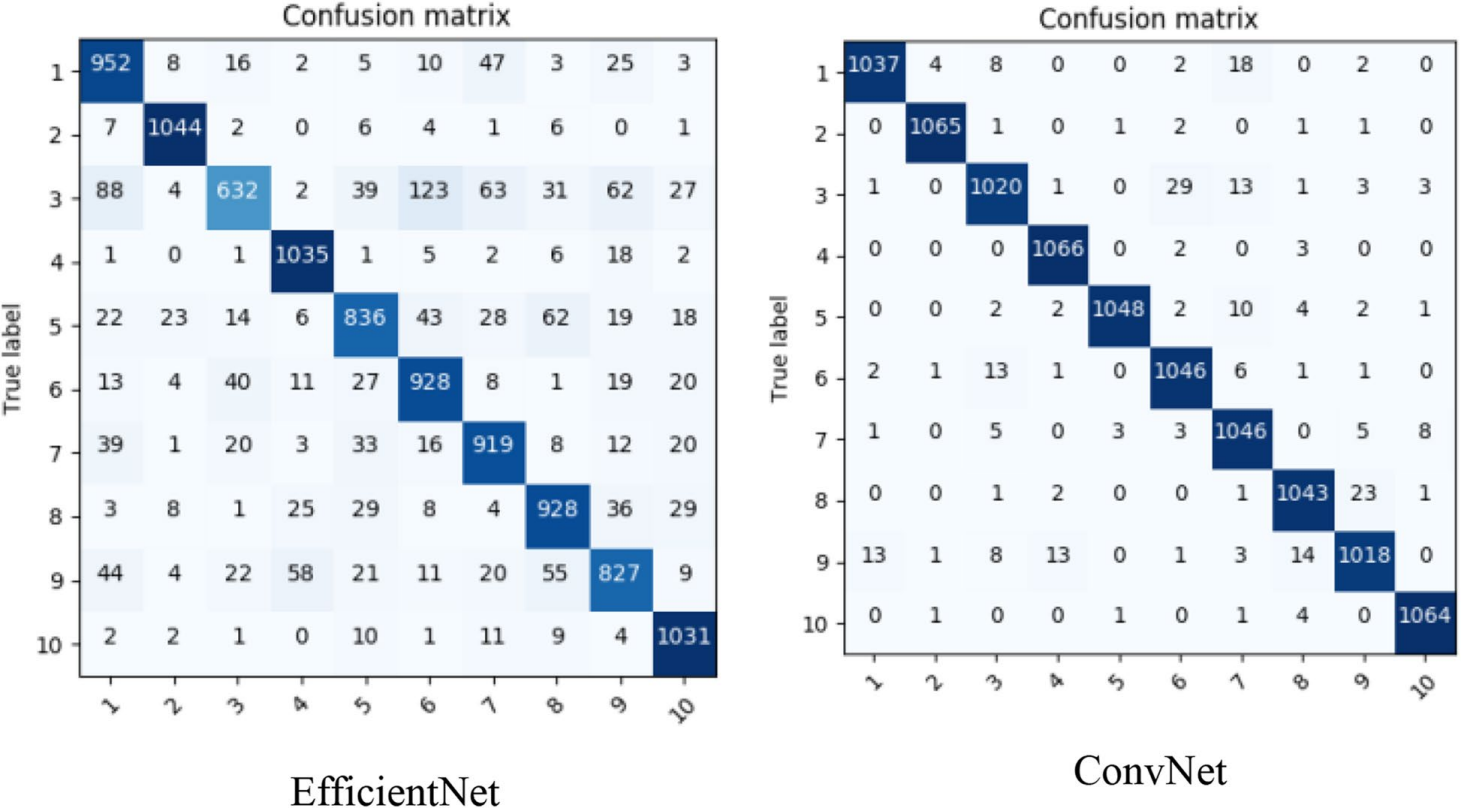
Tomato leaves (PlantVillage) classification results after replace layer replacement

**Fig. 6:**
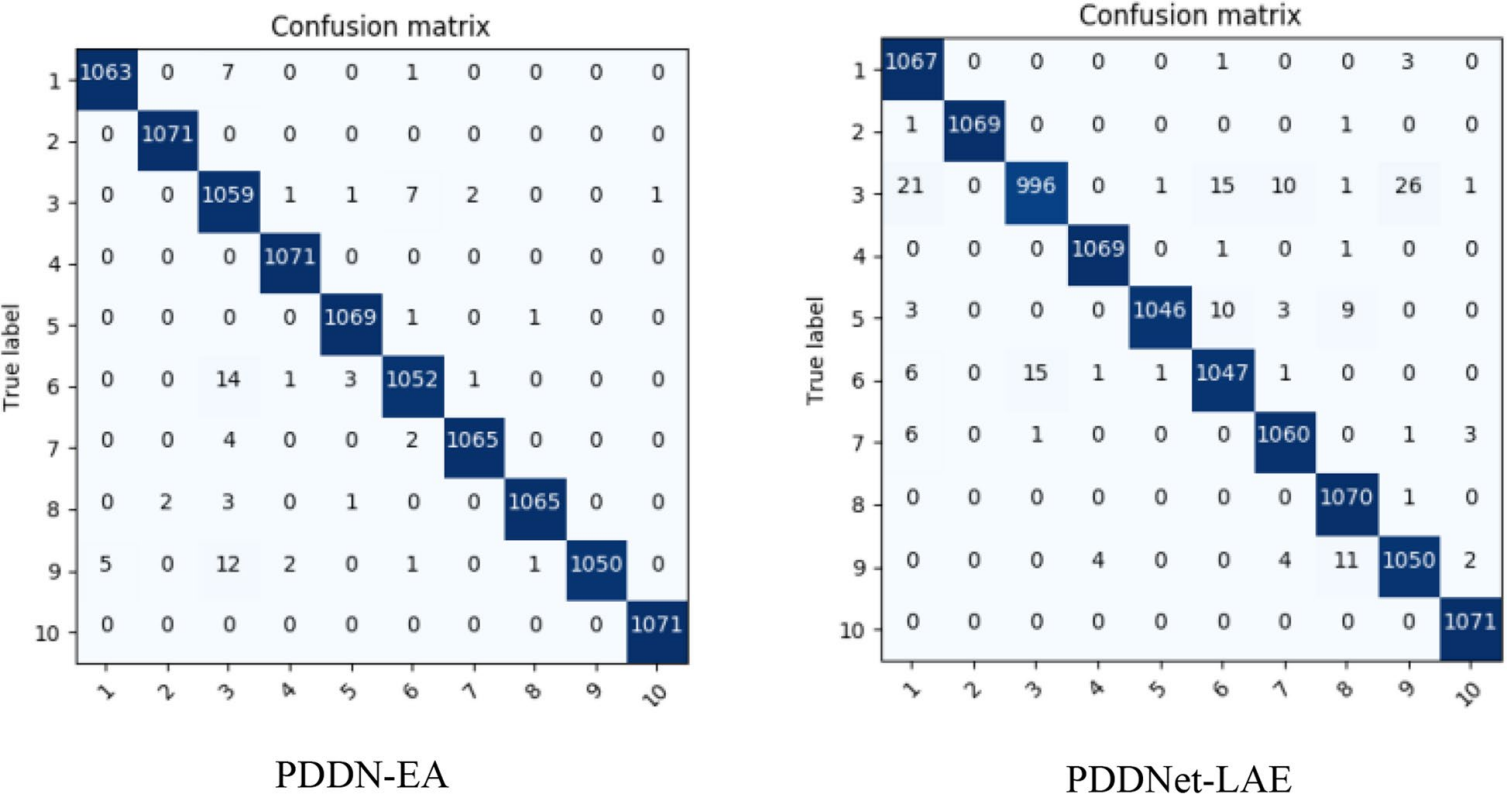
Tomato leaves (PlantVillage) classification results by proposed models classification

The study considers tomato class with 16,703 plant images obtained from the PlantVillage dataset entailing 1,591 healthy leaves, 373 *Mosaic Virus*, 3,209 *Yellow Leaf Curl Virus*, 1,404 *Target Spot*, 1,676 *Spider Mites Two Spotted Spider Mite*, 1,771 *Septoria leaf spot*, 952 *Leaf Mold*, 1,909 *Late Blight*, 1,000 *Late Blight*, 2,127 *Bacterial Spots* images as presented within the dataset. After 10 epochs, the classification results are demonstrated in Fig. 4, utilizing some of the considered pre-trained models, namely ResNet101 and DesnseNet201. Figure 5 presents a confusion matrix after replacing the first and second modified layers (i.e., a fully connected and a softmax layer) of EfficientNet and ConvNet. Figure 6 presents a confusion matrix of the proposed two models. Note: 1 through 10 on the horizontal axis depict the ten tomato leaf image categories.

## Conclusion

In this research, early fusion and lead voting ensembles were introduced, combined with nine pretrained CNNs, and fine-tuned for deep feature extraction. Using TL and 15 classes of PlantVillage Dataset, the models outperformed CNNs in plant and disease detection with 96.74% and 97.79% accuracy. These models are robust and generalizable, providing practical solutions to improve plant disease detection and classification accuracy and effectiveness, improving agricultural practices and sustainable food production as the population grows. The research's findings emphasize the significance of advanced technology in mitigating concerns associated with plant disease classification and detection.

In future research, focus on resolving issues related to real-time data collecting and creating a multi-object deep learning model capable of identifying plant illnesses based on a cluster of leaves rather than just a single leaf amidst comparative statistical analysis. Moreover, we are striving to implement a mobile application or web-enabled service utilizing the trained model derived from this research to support a wider plant disease research community to benefit the agricultural sector. Also, to move toward a more lightweight disease classification, model quantization, and object localization networks are critical to better spot the species leaves in a complex background using the trending vision transformers.

## Data Availability

The datasets generated during and analyzed during the current study are available at: https://www.kaggle.com/datasets/abdallahalidev/plantvillage-dataset.

## Footnotes

1 https://www.mathworks.com/products/deep-learning.html

2 https://www.mathworks.com/

3 https://www.nvidia.com/en-us/
